# Anti-inflammatory and Antioxidant Effects of Flavonoid-Rich Fraction of Bergamot Juice (BJe) in a Mouse Model of Intestinal Ischemia/Reperfusion Injury

**DOI:** 10.3389/fphar.2016.00203

**Published:** 2016-07-15

**Authors:** Daniela Impellizzeri, Marika Cordaro, Michela Campolo, Enrico Gugliandolo, Emanuela Esposito, Filippo Benedetto, Salvatore Cuzzocrea, Michele Navarra

**Affiliations:** ^1^Department of Chemical, Biological, Pharmaceutical and Environmental Sciences, University of Messina Messina, Italy; ^2^Department of Vascular and Thoracic Surgery, University of Messina Messina, Italy; ^3^Manchester Biomedical Research Centre, Manchester Royal Infirmary, School of Medicine, University of Manchester Manchester, UK

**Keywords:** bergamot juice, inflammation, oxidative stress, ischemia, cytokines, *Citrus bergamia*

## Abstract

The flavonoid-rich fraction of bergamot juice (BJe) has demonstrated anti-inflammatory and antioxidant activities. The aim of work was to test the beneficial effects of BJe on the modulation of the ileum inflammation caused by intestinal ischemia/reperfusion (I/R) injury in mice. To understand the cellular mechanisms by which BJe may decrease the development of intestinal I/R injury, we have evaluated the activation of signaling transduction pathways that can be induced by reactive oxygen species production. Superior mesenteric artery and celiac trunk were occluded for 30 min and reperfused for 1 h. The animals were sacrificed after 1 h of reperfusion, for both histological and molecular examinations of the ileum tissue. The experimental results demonstrated that BJe was able to reduce histological damage, cytokines production, adhesion molecules expression, neutrophil infiltration and oxidative stress by a mechanism involved both NF-κB and MAP kinases pathways. This study indicates that BJe could represent a new treatment against inflammatory events of intestinal I/R injury.

## Introduction

Intestinal IR is a phenomenon associated to physiological or pathophysiological events. Reperfusion of the ischemic intestine triggers the release of pro-inflammatory mediators that can intensify the injury (Campolo et al., 2013). SAO shock-mediated injury is an animal model of intestinal ischemia (Campolo et al., 2013). It causes local and systemic changes derived from the release of cytotoxic elements and from neutrophils and endothelial cells communication (Stefanutti et al., 2005). In severe cases, it causes bleeding, symptomatic intestinal stenosis, bowel perforation and peritonitis (Masini et al., 2006). Certain events during intestinal ischemia could subject the intestine to an augmented oxidative stress, with following activation of polymorphonuclear neutrophils (PMNs), ROS, RNS (Moore, 1997) production and activation of the redox-sensitive transcription factors, such as nuclear factor (NF-κB) and MAPK superfamily (Chen et al., 2004; Campolo et al., 2013).

*Citrus bergamia* Risso et Poiteau (bergamot) is a tree belonging to the Rutaceae family (subfamily Esperidea) cultivated in the southern coast of Calabria region (south of Italy). Bergamot fruit is mostly used to produce essential oil (BEO) which falls into the composition of many perfumes and is also used in aromatherapy (Navarra et al., 2015b). BEO has been traditionally utilized in Italian folk medicine for topical use as antiseptics for disinfection action and as aids for healing minor wounds (Meineri, 1952). Additionally, BEO has been investigated for its potential neuroprotective (Corasaniti et al., 2007) and anticancer effects (Celia et al., 2013; Navarra et al., 2015a). Bergamot juice (BJ) is obtained by squeezing the endocarp of the fruits, and recently has increased attention for its biological actions. It is known that flavonoids are efficient in inflammatory disease, cancer (Garcia-Lafuente et al., 2009) and dermal damage (Sannigrahi et al., 2011). Several mechanisms have been suggested, such as the suppression of COX-2 expression (Mutoh et al., 2000), decrease of ROS (Lee et al., 2002), the modulation of signaling pathways and down-regulation of NF-κB (Vidya Priyadarsini et al., 2010). Recently, the potential anti-tumor capacity of BJ has been exhibited both in *in vitro* (Delle Monache et al., 2013) and *in vivo* (Navarra et al., 2014) models, indicating that BJ may act by different mechanisms depending on the cell lines (Ferlazzo et al., 2016a). Moreover, it has been suggested that the flavonoids present in the BJ are responsible for its antiproliferative effect, by inducing apoptosis that in turn causes inhibition of human colon cancer cell growth (Visalli et al., 2014). Furthermore, the flavonoid-rich fraction of BJ (BJe) possesses antioxidant properties (Ferlazzo et al., 2015, 2016b) and counteracts inflammatory response (Risitano et al., 2014; Currò et al., 2016). The anti-inflammatory activity of BJe was also demonstrated in an experimental model of bowel disease (Impellizzeri et al., 2014) suggesting a possible role in treating inflammatory processes because it’s favorable balance between safety and efficacy (Marino et al., 2015).

The objective of the study, based on these findings, was to evaluate the beneficial outcomes of BJe in an experi mental model of intestinal ischemia caused by SAO in mice.

## Materials and Methods

### The Flavonoid-Rich Fraction of Bergamot Juice (BJe)

The name of the plant studied in this work “*Citrus bergamia* Risso and Poiteau” has been checked with The Plant list. This name was in version 1 of The Plant List, (record kew-2724031) and now is a synonym *of Citrus limon* (L.) Osbeck^1^.

BJe was a kindly gift offered by the company “Agrumaria Corleone” (Palermo, Italy) which took fruits of *Citrus bergamia* Risso and Poiteau from crops present in Reggio Calabria (Italy). BJe was centrifuged at 6000 rpm/min for 15 min to eliminate impurities and converted into a dry powder (spray drying method). Aliquots of BJe were stored at -20° C. The drug was defrosted and dissolved in saline before to use. The chemical profile of flavonoid-rich fraction of BJ has been previously described (Risitano et al., 2014). The major flavonoids found in BJ (mg/g) were neohesperidin (105.27), naringin (101.88), melitidin (75.89), neoeriocitrin (56.61), hesperetin (55.65) and naringenin (43.22).

### Animals

Adult CD1 mice (male) (25–30 g, 8–9 weeks Harlan, Italy) were kept in a well organized environment with standard rodent chow and water. Animals were accommodated in stainless steel cages into a room at 22 ± 1°C with a 12-h light, 12-h dark cycle. The study was permitted by the University of Messina Review Board for the care of animals. All animal experiments were performed following the regulations in Italy (D.M. 116192), Europe (O.J. of E.C. L 358/1 12/18/1986), USA (Animal Welfare Assurance No A5594-01, Department of Health and Human Services, USA).

### Induction of SAO

The induction of SAO was performed as previously described from our studies (Campolo et al., 2013).

Specifically, after anesthesia, SAO model was induced in mice by clamping the superior mesenteric artery and the celiac trunk, for 30 min following 1 h of reperfusion. After the reperfusion period, animals were killed for histological, immunohistochemical, biochemical and western blots analyses of ileum tissues.

In another set of experiments, following reperfusion, the various groups (*n* = 10 for each group) of mice were observed for 24 h to evaluate survival differences.

### Experimental Groups:

Mice were in random divided in different groups:

(1) I/R + vehicle group: mice (*n* = 10) were subjected to intestinal ischemia by SAO (30 min), followed by reperfusion (1 h) plus administration of vehicle (saline).
(2) I/R + BJe group: mice were subjected to surgical procedures, described as above, and BJe was administered intraperitoneally (20 mg/kg) 5 min prior to reperfusion (*n* = 10).
(3) Sham + vehicle group: mice were subjected to surgical procedures except for SAO shock and were kept under anesthesia for the time of the experiment (*n* = 10) plus administration of vehicle (saline).
(4) Sham + BJe group: equal to sham-operated mice, except for the administration of BJe (20 mg/kg i.p.), 5 min prior to reperfusion (*n* = 10).

The dose of BJe (20 mg/kg) used here was based on previous *in vivo* study (Impellizzeri et al., 2014).

### Light Microscopy

Histological examination was performed as indicated in our previous studies (Cuzzocrea et al., 2004). For quantitative estimation of I/R injury, sections (*n* = 10 for each animal) were examined by two independent observers blinded to the experimental protocol. The morphological criteria were used as described previously (Cuzzocrea et al., 2004): score 0, no damage; score 1 (mild), focal epithelial edema and necrosis; score 2 (moderate), diffuse swelling and necrosis of the villi; score 3 (severe), necrosis with presence of neutrophil infiltrate in the submucosa; score 4 (highly severe), widespread necrosis with massive neutrophil infiltrate and hemorrhage.

### Myeloperoxidase Activity

Myeloperoxidase activity is an indicator of PMN accumulation and was determined as described previously (Mullane, 1989).

### Measurement of Cytokines

Tumor necrosis factor (TNF)-α and interleukin (IL)-1β levels were measured in ileum samples as described from our previous study (Campolo et al., 2013).

### Malondialdehyde Measurement

Malondialdehyde levels were measured as an indicator of lipid peroxidation as indicated from previous our study (Campolo et al., 2013).

### Immunohistochemical Localization of Intercellular Adhesion Molecule (ICAM)-1 and P-Selectin

Immunohistochemical analysis was performed as described previously (Campolo et al., 2013).

The sections were incubated overnight with primary anti-P-selectin antibody (BD PharMingen, San Diego, CA, USA; CD62P, 1:500), and anti-ICAM-1 antibody (BD PharMingen; CD54, 1:500), or control solutions. Controls included buffer alone or non-specific-purified rabbit IgG. Sections were washed with PBS, incubated with secondary antibody. Specific labeling was detected with a biotin-conjugated goat anti-rabbit IgG and avidin–biotin peroxidase complex (Vector Laboratories, Burlingame, CA, USA). Immunohistochemistry photographs were assessed by densitometry by using (Leica QWin V3, UK).

### Western Blots for IkB-α, NF-κBp65, p-JNK, p-P38, Inducible Nitric Oxide Synthase, Manganese Superoxide Dismutase

Western blots were performed as described from our previous studies (Campolo et al., 2013).

Specific primary antibodies, anti-IkB-α (1:500; Santa Cruz Biotechnology), anti-NF-κB p65 (1:500; Santa Cruz Biotechnology), anti- p-JNK (1:500; Santa Cruz Biotechnology) anti-iNOS (1:500; BD Transduction), anti-p-P38 (1:1000; Cell signaling) and anti-MnSOD (1:500 Millipore) in 1x PBS, 5%(w/v) non-fat dried milk, and 0.1% Tween 20 were used at 4°C overnight. Membranes were incubated with peroxidase-conjugated bovine anti-mouse IgG secondary antibody or peroxidase-conjugated goat anti-rabbit IgG (1:2000; Jackson Immuno Research Laboratories, West Grove, PA, USA) for 1 h at room temperature. The levels of β-actin (1:2000; Santa Cruz Biotechnology) and lamin A/C (nuclear fraction 1:500 Sigma–Aldrich, Milan, Italy) served as an internal control for protein loading. The relative expression of the protein bands of IkB-α (37 kDa), NF-κB p65 (65 kDa), iNOS (130 kDa), p-JNK (46 kDa), p-P38 (38 kDa), MnSOD (24 Kda) were detected with an enhanced chemiluminescence (ECL) system (Thermo, USA) and visualized with the Chemi Doc XRS (Bio-Rad, USA) and analyzed by using Image Lab 3.0 software (Bio-Rad, USA).

### Materials

All substances were from Sigma–Aldrich (Milan, Italy). All stock solutions were dissolved in non-pyrogenic saline (0.9% NaCl; Baxter, Italy, UK).

### Statistical Evaluation

Data are expressed as mean ± SEM of N observations (the number of animals). For histology or immunohistochemistry analyses, the presented figures are representative of at least three independent experiments. The results were analyzed by one way ANOVA followed by a Bonferroni *post hoc* test for multiple comparisons. A *p*-value of less than 0.05 was considered significant.

## Results

### Effects of BJe on Histological Damage Induced by Gut I/R

**FIGURE 1:**
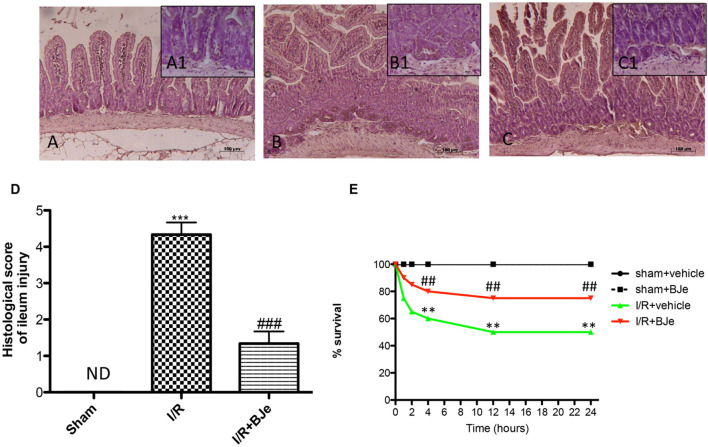
**Effect of BJe on histological alteration of ileum tissue, and on survival rate**. Histological features of normal gut tissue were found in ileum tissues from sham-operated mice (**A,A1**; see Histological Score, **D**). Distal ileum section from I/R-subjected mice showed inflammatory infiltration by PMNs, concentrated below the epithelial layer and edema of the distal portion of the villi (**B,B1**; see Histological Score, **D**). BJe treatment reduced I/R-induced organ injury (**C,C1**; see Histological Score, **D**). The figures are representative of at least three experiments performed on different experimental days. Survival was monitored for 24 h after SAO shock **(E)**. BJe administration is able to reduce the mortality induced by I/R injury. Data are means ± SEM of 10 mice for each group. ^∗∗^ *P* < 0.01 vs. SHAM; ^∗∗∗^ *P* < 0.001 vs. SHAM; ^##^ *P* < 0.01 vs. I/R; ^###^ *P* < 0.001 vs. I/R. ND, not detectable.

The ileum of mice subjected to I/R showed severe histological alteration with edema of intestinal villi (**Figures 1B,B1**; see Histological Score **Figure 1D**). BJe (20 mg/kg) treatment reduced histological damage (**Figures 1C,C1**; see Histological Score, **Figure 1D**). The histological structure of the gut tissue from sham mice was as a normal architecture (**Figures 1A,A1**; see Histological Score, **Figure 1D**).

### Effects of BJe on Survival Rate

Sham mice survived the entire 24 h reperfusion period. In contrast, intestinal I/R caused a shock state characterized by 60% mortality at the end of reperfusion (**Figure 1E**). BJe administration was able to decrease the mortality induced by I/R (**Figure 1E**).

### Effects of BJe on Cytokines Production and Neutrophil Infiltration in Ileum Tissue

**FIGURE 2:**
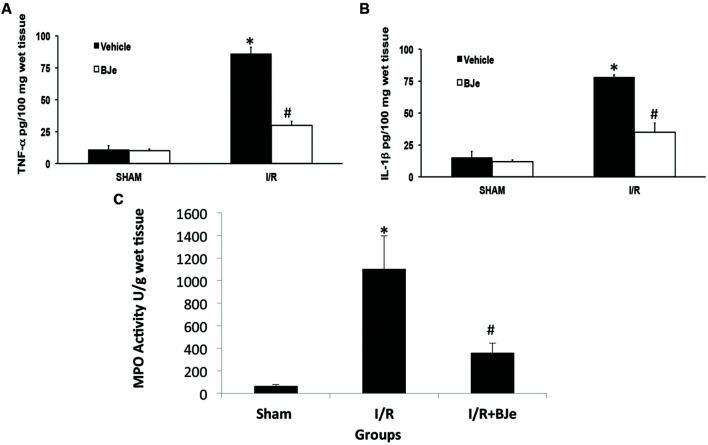
**Effect of BJe on cytokine release and neutrophil infiltration**. TNF-α and Il-1β ileum levels were significantly elevated after 1 h of reperfusion in I/R subjected mice **(A,B)** compared to sham mice **(A,B)**. BJe reduced TNF-α and IL-1β levels **(A,B)**. In addition, neutrophil infiltration was measured by MPO assay. Increased MPO activity was found in I/R subjected mice compared to sham animals **(C)**. BJe treatment was able to decrease MPO activity in a significant way **(C)**. Data are means ± SEM of 10 mice for each group. ^∗^*P* < 0.05 vs. SHAM; ^#^*P* < 0.05 vs. I/R.

An increase in the levels of TNF-α and IL-1β in ileum samples was found in I/R subjected mice compared to sham group (**Figures 2A,B**). The levels of TNF-α and IL-1β were attenuated by treatment with BJe (**Figures 2A,B**). In addition, neutrophil infiltration was evaluated by ileum MPO assay. The treatment with BJe significantly decreased neutrophil infiltration into the intestinal tissue compared to vehicle group (**Figure 2C**).

### Effects of BJe on Oxidative Stress

**FIGURE 3:**
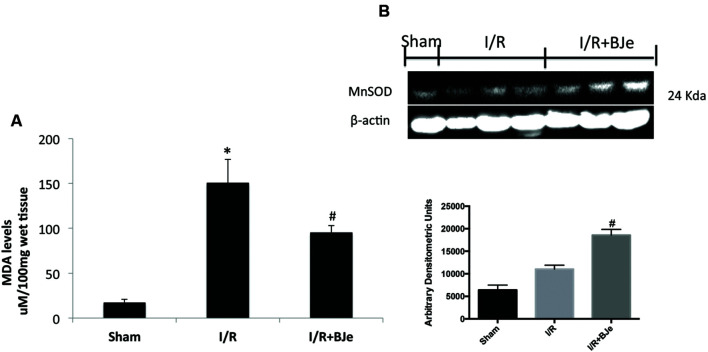
**Effect of BJe on MDA levels and MnSOD expression**. Reperfusion of the ischemic splanchnic circulation leads to a profound increase in MDA levels in ileum tissues from I/R subjected mice treated with vehicle **(A)**. MDA levels were reduced by BJe treatment **(A)**. In addition, the expression of an antioxidant enzyme MnSOD, was also evaluated by western blot **(B)**. The expression of MnSOD was increased in BJe treated mice compared to vehicle group **(B)**. Basal expression was found in sham mice **(B)**. A representative blot of lysates **(B)** obtained from 10 animals/group is shown, and densitometry analysis of all animals is reported. Data are means ± SEM of 10 mice for each group. ^∗^*P* < 0.05 vs. SHAM; ^#^*P* < 0.05 vs. I/R.

Ischemia/reperfusion caused an increase in lipid peroxidation in ileum tissues. MDA levels were detected in the ileum tissues as an indicator of lipid peroxidation. A significant increase in MDA levels (**Figure 3A**) was observed in mice subjected to intestinal I/R compared to sham-treated mice. MDA levels were significantly attenuated by BJe (**Figure 3A**). In addition, to test whether BJe could modulate the oxidative process we also analyzed the expression of antioxidant enzyme MnSOD. A basal expression of MnSOD was observed in the colon tissues from sham-treated mice (**Figure 3B**). BJe treatment (20 mg/kg) significantly increased MnSOD expression compared to I/R mice (**Figure 3B**).

### Effects of BJe on ICAM-1 and P-Selectin Expression

**FIGURE 4:**
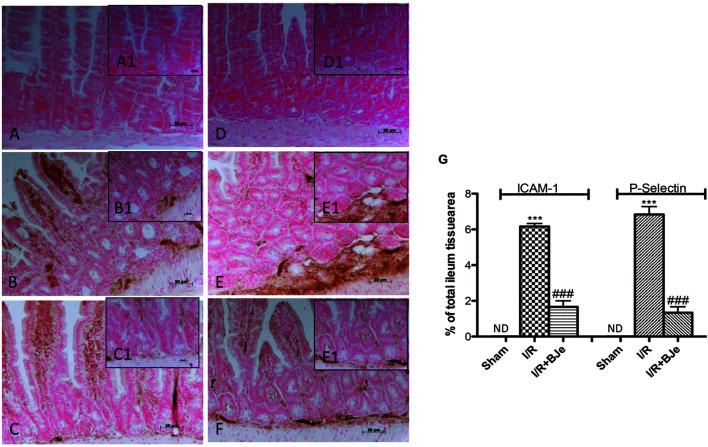
**Effect of BJe on adhesion molecules expression**. Ileum sections taken from I/R subjected- mice showed positive staining for ICAM-1 **(B,B1,G)** and for P-selectin **(E,E1,G)** compared to sham-operated mice **(A,A1,D, D1,G)**. The degree of positive staining for adhesion molecules was reduced in tissue sections from mice treated with BJe **(C,C1,F,F1,G)**. The figures are representative of at least three experiments performed on different experimental days. Data are indicated as means ± SEM of 10 mice for each group. ^∗∗∗^*P* < 0.001 vs. SHAM; ^###^*P* < 0.001 vs. I/R. ND, not detectable.

I/R was characterized by increased imununohistochemical positive staining of adhesion molecules, such as ICAM-1 and P-selectin in the ileum sections obtained from injured mice mainly localized around the vessels (**Figures 4B,B1,E,E1**; see densitometric analysis, **Figure 4G**). The positive staining in the ileum tissue for ICAM-1 and P-selectin (**Figures 4C,C1,F,F1**; see densitometric analysis, **Figure 4G**) was reduced by BJe treatment. No positive staining for ICAM-1 and P-selectin was observed in the ileum sections from sham group (**Figures 4A,A1,D,D1**; see densitometric analysis, **Figure 4G**).

### Effects of BJe on IκB-α Degradation, NF-κB p65 Translocation and iNOS Expression

**FIGURE 5:**
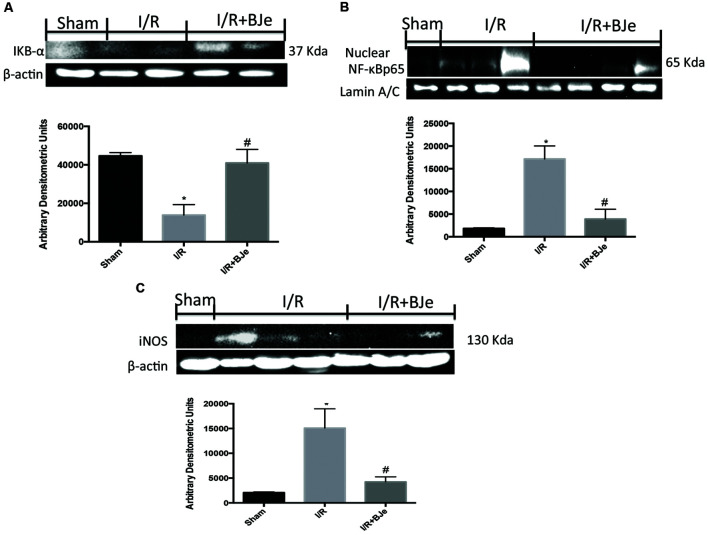
**Effect of BJe on NF-κB pathway**. Representative western blots showing the effects of BJe on IκB-α degradation **(A)**, NF-κB p65 translocation **(B)** and iNOS expression **(C)** after SAO shock. BJe treatment reduced IκB-α degradation **(A)**, NF-κB p65 translocation **(B)** and iNOS expression **(C)**. A representative blot of lysates **(A,B)** obtained from 10 animals/group is shown, and densitometry analysis of all animals is reported. The results in **(A–C)** are expressed as means ± SEM of 10 mice for each group. ^∗^*P* < 0.05 vs. SHAM; ^#^*P* < 0.05 vs. I/R.

Basal levels of IκB-α were detected in the ileum from sham mice (**Figure 5A**), whereas the levels of IκB-α were reduced in mice subjected to I/R (**Figure 5A**). BJe treatment was able to prevent the I/R –induced IκB-α degradation (**Figure 5A**). Likewise, p65 subunit translocation protein in the nuclei of the ileum tissue was amplified after intestinal I/R compared to sham mice (**Figure 5B**). BJe administration significantly reduced the translocation of p65 in nuclear homogenates (**Figure 5B**). In addition, we also evaluated the expression of iNOS that is under the control of NF-κB pathway. I/R subjected mice showed an increased expression of iNOS compared to sham animals (**Figure 5C**). Moreover, BJe treatment was able to reduce this expression in a significant way (**Figure 5C**).

### Effects of BJe on MAP Kinases Pathway

**FIGURE 6:**
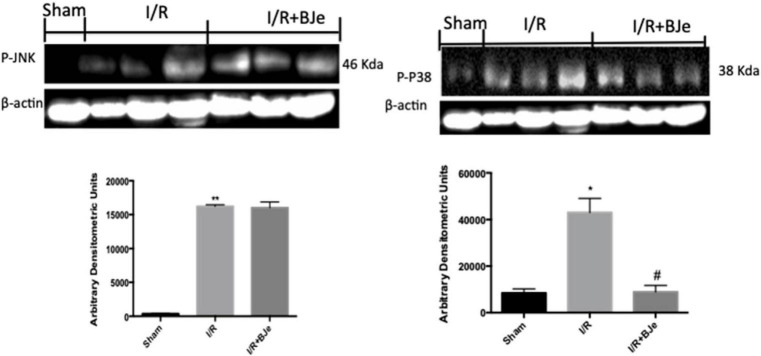
**Effect of BJe on MAPKs pathway.** Representative western blots showing the effects of BJe on p-JNK **(A)**, and p-P38 expression **(B)** after I/R injury. I/R caused an increase in p-JNK **(A)**, and p-P38 **(B)** expression. BJe treatment was not able to reduce p-JNK **(A)** but significantly decreased p-P38 expression **(B)**. A representative blot of lysates **(A, B)** obtained from 10 animals/group is shown, and densitometry analysis of all animals is reported. The results in **(A, B)** are expressed as means ± SEM of 10 mice for each group. *P < 0.05 vs. SHAM; ^**^P < 0.01 vs. SHAM; ^#^*P* < 0.05 vs. I/R.

To better understand the mechanism of action of BJe treatment, we also analyzed by western blot MAP kinases pathway activation by the expression of p-JNK and p-P38. I/R caused an increased p-JNK and p-P38 expression in ileum tissues obtained from mice subjected to I/R + vehicle (**Figures 6A,B**). Moreover, BJe treatment (20 mg/kg) was not able to attenuate p-JNK expression in a significant way (**Figure 6A**). On the contrary, the expression of p-P38 was markedly reduced by BJe treatment (**Figure 6B**).

## Discussion

Bergamot, a typical fruit of the Reggio Calabria province in Southern Italy, is known for its essential oil obtained from the peel, that is employed in the cosmetic, pharmaceutical and food industry (Verzera et al., 2003; Navarra et al., 2015a). Compared to other *Citrus* juice, because of its bitter taste, the BJ is considered a byproduct of the essential oil production, scarcely employed by the food industry. The beneficial effects of BJ have been increasing interest for its health promoting activity (Mollace et al., 2011; Graziano et al., 2012; Impellizzeri et al., 2014; Navarra et al., 2014; Risitano et al., 2014; Visalli et al., 2014; Filocamo et al., 2015; Currò et al., 2016).

Intestinal injury as a result of I/R is a life risk and clinical emergency. The interruption of blood supply produces ischemia, which can damage metabolically active tissues. The return of blood flow after a period of ischemia is important to maintain cell function. However, restoration of blood flow to the ischemic tissues causes pathophysiologic responses that lead to additional cell and tissue injury (Di Paola et al., 2012). Several mediators, such as ROS, proinflammatory cytokines and chemokines contribute markedly to the degree of the ileum injury (Paterniti et al., 2010).

The aim of study was to investigate the antioxidant and anti-inflammatory effects of a BJe on the pathophysiological events induced by a mouse model of intestinal I/R (SAO model). In particular, we observed that ileum section from SAO mice shown inflammatory infiltration by PMNs with edema of the villi. The treatment with BJe was able to reduce the histological damage in ileum tissues.

To understand the cellular mechanisms by which BJe may decrease the development of intestinal I/R injury, we have evaluated the activation of signaling transduction pathways that can be induced by ROS production, such as NF-κB and MAPKs. During inflammatory conditions, ROS can regulate phagocytosis and production of neutrophil-derived ROS, and may influence endothelial and epithelial cells contributing to the intensification of tissue injury. Furthermore, oxidative stress stimulates the activation of the redox-sensitive transcription factors, such as NF-κB, which have a central action in the expression of inflammatory cytokines, adhesion molecules and the activation of the redox sensitive protein kinases, the MAPK superfamily (Campolo et al., 2013). We showed here that intestinal I/R results in an increase of p-JNK, p-P38 expression, NF-κB translocation and IKB-α degradation in the ileum inflamed tissues, whereas BJe significantly reduced p-P38 expression, NF-κB translocation and inhibited the IκB-α degradation. Interestingly, previous study demonstrated that BJe reduced gene expression as well as the levels of LPS induced pro-inflammatory cytokines (IL-6, IL-1β, TNF-α) in THP-1 monocytes by a mechanism involving the inhibition of NF-κB (Risitano et al., 2014). More recently we demonstrated that BJe modulated NF-κB and MAPKs pathways in a mouse model of colitis induced by intracolonic instillation of dinitrobenzene sulfonic acid (Impellizzeri et al., 2014). Furthermore, we also observed that gut I/R caused an increase of TNF-α and IL-1β levels and the formation of P-selectin on the endothelial vascular wall and ICAM-1 expression on endothelial cells. Treatment with BJe inhibited cytokines production and the expression of P-selectin and ICAM-1, as already observed in other experimental model (Kim et al., 2011; Impellizzeri et al., 2014). The reduction of the expression of the adhesion molecules could be related with the reduction of infiltration of leucocytes and attenuation of the ileum tissue injury in mice treated with BJe. Activation of leukocytes resulted in ROS release that during reperfusion can react with nitrogen species to produce oxygen-free radicals, $O_{2}^{-}$ and H_2_O_2_. The damaging effects of $O_{2}^{-}$, under physiological conditions, are prevented by SOD, which transforms $O_{2}^{-}$ to H_2_O_2_. However, during reperfusion of ischemic tissues, these natural defenses may be destroyed (Salvemini and Cuzzocrea, 2002; Paterniti et al., 2010). In this work, we observed that BJe treatment was able to inhibit nitrosative and oxidative stress by reducing lipid peroxidation (MDA levels), iNOS production and by increasing antioxidant such as MnSOD enzyme. In that regard, recent results demonstrated the antioxidant properties of BJe in both abiotic and *in vitro* experimental models (Ferlazzo et al., 2015). The research documented that the flavonoids present in BJe reduces both the generation of ROS and lipid peroxidation, improves the functionality of mitochondria and prevents DNA-oxidative injury in A549 cells treated with H_2_O_2_ (Ferlazzo et al., 2015).

Our study reported that BJe was able to reduce the secondary injury associated to intestinal damage, supporting the role of bergamot flavonoids as nutraceuticals or functional food useful to protect against inflammatory intestinal diseases such as gut I/R injury.

In summary, in this study, BJe treatment could inhibit intestinal inflammation caused by I/R injury by reducing: ROS/RNS production – inflammatory NF-κB and MAPKs pathways – pro-inflammatory cytokines levels and neutrophil infiltration – adhesion molecules expression – oxidative and nitrosative stress – tissue injury.

Until today, there is less information in the literature about the protective effects and possible mechanism of action of BJe on intestinal inflammatory disorders. The present study could provide a scientific basis for the use of BJe as a popular medicine and could better elucidate its possible mechanism of action using *in vivo* models.

## Author Contributions

DI drafted the manuscript, participated in research design and carried out the *in vivo* experiments. EG carried out the *in vivo* experiments; MrC and McC performed immunohistochemical analysis and performed western blot analysis; EE and FB analyzed the data; SC, EE, and MN contributed to the writing of the manuscript and designing the experiments. All authors read and approved the final manuscript.

## Conflict of Interest Statement

Agrumaria Corleone had no role in study design, data collection and analysis, decision to publish, or preparation of the paper and the authors declare that the research was conducted in the absence of any commercial or financial relationships that could be construed as a potential conflict of interest. The reviewer VB and DT, and handling Editor declared their shared affiliation, and the handling Editor states that the process nevertheless met the standards of a fair and objective review.

## Footnotes

1
http://www.theplantlist.org/tpl1.1/record/tro-28101295

## ABBREVIATIONS

BJ: bergamot juice
BJe: flavonoid-rich fraction of bergamot juice
COX-2: cyclooxygenase-2
ICAM: intercellular adhesion molecule
IL: interleukin
iNOS: inducible nitric oxide synthase
IR: ischemia-reperfusion
MAPK: mitogen activated protein kinases
MDA: malondialdehyde
MnSOD: manganese superoxide dismutase
MPO: myeloperoxidase
NF: nuclear factor
RNS: reactive nitrogen species
ROS: reactive oxygen species
SAO: splanchnic artery occlusion
TNF: tumor necrosis factor
