# Corrigendum: Anti-inflammatory and antioxidant effects of flavonoid-rich fraction of bergamot juice (BJe) in a mouse model of intestinal ischemia/reperfusion injury

**DOI:** 10.3389/fphar.2024.1434215

**Published:** 2024-07-05

**Authors:** Daniela Impellizzeri, Marika Cordaro, Michela Campolo, Enrico Gugliandolo, Emanuela Esposito, Filippo Benedetto, Salvatore Cuzzocrea, Michele Navarra

**Affiliations:** ^1^ Department of Chemical, Biological, Pharmaceutical and Environmental Sciences, University of Messina, Messina, Italy; ^2^ Department of Vascular and Thoracic Surgery, University of Messina, Messina, Italy; ^3^ Manchester Biomedical Research Centre, Manchester Royal Infirmary, School of Medicine, University of Manchester, Manchester, United Kingdom

**Keywords:** bergamot juice, inflammation, oxidative stress, ischemia, cytokines, *Citrus bergamia*

In the published article, there was an error in Figure 6 as published. During the editing of the figures, the authors performed a factual error in the figures, copying and pasting the same band. The corrected Figure 6 appears below.

**FIGURE 6:**
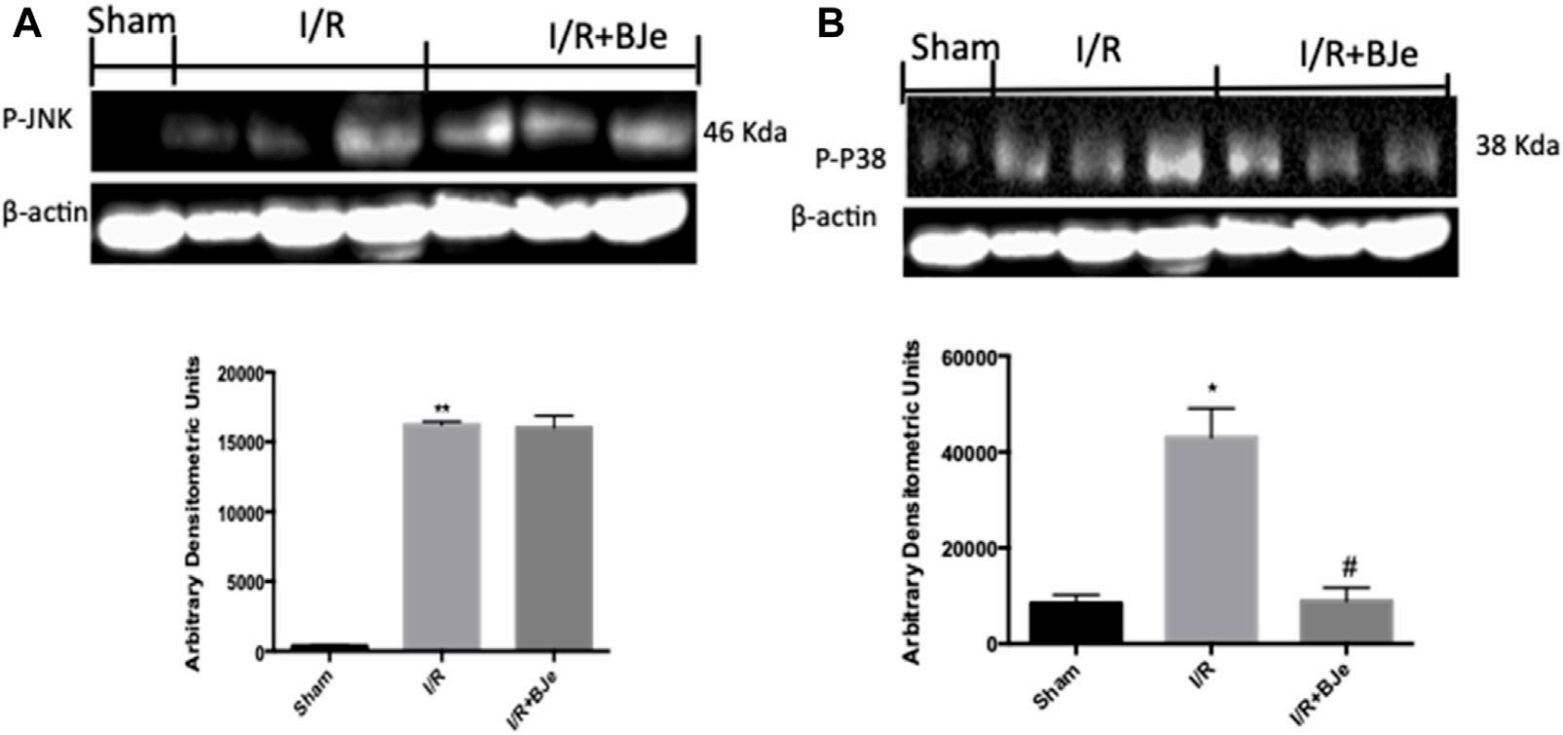
Effect of BJe on MAPKs pathway. Representative western blots showing the effects of BJe on p-JNK **(A)**, and p-P38 expression **(B)** after I/R injury. I/R caused an increase in p-JNK **(A)**, and p-P38 **(B)** expression. BJe treatment was not able to reduce p-JNK **(A)** but significantly decreased p-P38 expression **(B)**. A representative blot of lysates **(A, B)** obtained from 10 animals/group is shown, and densitometry analysis of all animals is reported. The results in **(A, B)** are expressed as means ± SEM of 10 mice for each group. *p < 0.05 vs. SHAM; **p < 0.01 vs. SHAM; ^#^ *p* < 0.05 vs. I/R.

The authors apologize for this error and state that this does not change the scientific conclusions of the article in any way. The original article has been updated.

